# *Ex Vivo* Antioxidant Activity of Selected Medicinal Plants against Fenton Reaction-Mediated Oxidation of Biological Lipid Substrates

**DOI:** 10.1155/2015/728621

**Published:** 2015-12-31

**Authors:** Namratha Pai Kotebagilu, Vanitha Reddy Palvai, Asna Urooj

**Affiliations:** Department of Studies in Food Science and Nutrition, University of Mysore, Manasagangothri, Mysuru, Karnataka 570006, India

## Abstract

Free radical-mediated oxidation is often linked to various degenerative diseases. Biological substrates with lipids as major components are susceptible to oxygen-derived lipid peroxidation due to their composition. Lipid peroxide products act as biomarkers in evaluating the antioxidant potential of various plants and functional foods. The study focused on evaluation of the antioxidant potential of two extracts (methanol and 80% methanol) of four medicinal plants,* Andrographis paniculata*,* Costus speciosus, Canthium parviflorum*, and* Abrus precatorius*, against Fenton reaction-mediated oxidation of three biological lipid substrates; cholesterol, low-density lipoprotein, and brain homogenate. The antioxidant activity of the extracts was measured by thiobarbituric acid reactive substances method. Also, the correlation between the polyphenol, flavonoid content, and the antioxidant activity in biological substrates was analyzed. Results indicated highest antioxidant potential by 80% methanol extract of* Canthium parviflorum* (97.55%), methanol extract of* Andrographis paniculata* (72.15%), and methanol extract of* Canthium parviflorum* (49.55%) in cholesterol, low-density lipoprotein, and brain, respectively. The polyphenol and flavonoid contents of methanol extract of* Andrographis paniculata* in cholesterol (*r* = 0.816) and low-density lipoprotein (*r* = 0.948) and* Costus speciosus* in brain (*r* = 0.977, polyphenols, and *r* = 0.949, flavonoids) correlated well with the antioxidant activity. The findings prove the antioxidant potential of the selected medicinal plants against Fenton reaction in biological lipid substrates.

## 1. Introduction

Biological lipid substrates are prone to oxidation due to their composition. Oxygen-derived free radicals such as peroxyl radicals (ROO^•^) and hydroperoxyl radicals (HOO^•^) have a role in fatty acid peroxidation and have received great attention in connection with oxidative stress. The integrity of cell membranes is disturbed due to peroxidation leading to rearrangement of the membrane structure of lipid dense sites such as the brain, low-density lipoprotein (LDL), and cholesterol [1].

High levels of polyunsaturated fatty acids, oxygen utilization, and redox metal ions and relatively poor antioxidant systems make the brain tissue vulnerable to oxidative damage. Oxidative stress in the brain is linked to degenerative disease such as Alzheimer's disease (AD) wherein presence of increased levels of oxidative stress markers including the markers of lipid peroxidation such as acrolein, 4-hydroxy-2-trans-nonenal (HNE), and malondialdehyde (MDA) is observed. Brains of subjects with Mild Cognitive Impairment (MCI), arguably the earliest form of AD, show increased levels of MDA, free HNE, protein-bound HNE, F(2)-isoprostanes, F(4)-neuroprostanes, and acrolein. Thus, this highly oxidative environment is a hallmark of MCI and AD pathology [2].

The link between LDL and development of atherosclerosis is well known. The mechanism involves oxidation of LDL lipids and proteins leading to loss of recognition by the LDL receptor and a shift to recognition by scavenger receptors. The acetyl-LDL receptors on the macrophages take up these modified LDL and cholesterol; since the local concentration of cholesterol does not downregulate the activity of these receptors, thus projecting the molecular link between LDL, cholesterol, formation of foam cells, and development of atherosclerosis [3, 4].

The free radical-mediated oxidation of cholesterol gives 7*α*- and 7*β*-hydroperoxycholesterol, 7*α*- and 7*β*-hydrocholesterol; 5*α*, 6*α*-, and 5*β*,6*β*-epoxycholesterol, and 7-ketocholesterol as major products. Linoleates and cholesterol are abundant lipids* in vivo* and their free radical-mediated oxidation gives rise to hydroperoxy octadecadienoates (HPODEs) and 7-hydroperoxycholesterol. Oxysterols, products of cholesterol oxidation, serve as diagnostic biomarker of oxidative stress as intermediates in bile acid synthesis, messengers of cell signaling, and cholesterol transport [5].

The thiobarbituric acid (TBA) assay is the most commonly used assay to study lipid peroxidation. Addition of iron or copper salts to biological molecules causes site-specific formation of oxygen-derived free radicals such as peroxyl radical (ROO^•^) and hydroperoxyl radical (HOO^•^) which can trigger lipid peroxidation chain reactions. This reaction occurs by abstracting a hydrogen atom from a side chain methylene carbon of polyunsaturated fatty acids and transforms it into lipid hydroperoxides. These lipid hydroperoxides easily decompose to secondary products, such as aldehydes and MDAs, which can be detected by thiobarbituric acid reactive substances (TBARS) method [1, 4]. Level of lipid peroxidation products* in vivo* is determined by the balance between their formation, metabolism, secondary reactions, and excretion. These biomarkers are useful for evaluating the beneficial effects of antioxidant foods, spices, beverages, supplements, and drugs [6].

Several antioxidants protect biomolecules against oxidation. The protective effect of antioxidants and phytochemicals such as ubiquinol-10, ascorbate, lycopene, *β*-carotene, *α*-tocopherol [7, 8], flavonoids [9, 10], carotenoids, and phenolic compounds [11] against LDL oxidation; vitamins A, B_3_, C, and E [12, 13], anthocyanins [14], and quercetin [15] against brain oxidation; vitamin E and *γ*-oryzanol against cholesterol oxidation [16] has been reported.

Leaves of medicinal plants such as* Albizia amara*,* Achyranthes aspera*,* Cassia fistula*,* Cassia auriculata*, and* Datura stramonium* [17], bark of* Crataeva nurvala* [18], and root of* Curcuma longa* [19] have exhibited antioxidant activity against lipid oxidation.

In our laboratory, several medicinal plants of the Western Ghats, India, have been screened for their antioxidant efficacy by incorporating them into food systems such as biscuits and edible oil and in biological systems such as tissue substrates [20–26]. Furthermore, selected medicinal plants such as* Andrographis paniculata*,* Costus speciosus*,* Canthium parviflorum*, and* Abrus precatorius* were screened for their ability to inhibit oxidation in red blood cells and microsomes [27]. With this background, the present research work focused on the* ex vivo* antioxidant ability of the selected medicinal plants in biological lipid substrates such as cholesterol, LDL, and brain homogenate. Cholesterol is used as a model substrate in this experiment since it is one of the primary components of the brain and LDL.

## 2. Materials and Methods

### 2.1. Chemicals

All the chemicals used were of analytical grade. Protein kit was purchased from Span Diagnostics Ltd., Gujarat, India. LDL diagnostic kit was purchased from Agappe Diagnostics Ltd., Kerala, India.

### 2.2. Plant Materials

The plant samples selected for the study,* Andrographis paniculata* (AnP),* Costus speciosus *(CS)*, Canthium parviflorum* (CP), and* Abrus precatorius* (AP), were collected from the Western Ghats, India. The plant samples were identified by Botanist Dr. Janardhan, Department of Studies in Botany, University of Mysore, Mysuru, India. The leaves were cleaned, washed, and dried in hot air oven at 55°C for 8–10 h. The dried leaves were ground to a fine powder and passed through 60 mesh sieve and stored in airtight containers until further use.

### 2.3. Preparation of Extracts

Two different extracts were prepared from the dehydrated samples, that is, methanol (M) and 80% methanol (80M). 10 g of each sample was extracted with 100 mL of the solvent in a mechanical shaker for 12 hours and filtered through Whatman No. 1 filter paper. The filtrate obtained was evaporated to dryness in a Rotary evaporator at 50°C (Superfit, Bangalore, India). The extracts were stored at 4°C until further use.

### 2.4. Estimation of Polyphenol and Flavonoid Content in the Extracts

The polyphenol and flavonoid content was analyzed using Folin-Ciocalteu micromethod and pharmacopoeia method, respectively [28, 29]. The analysis was done on all samples of both the extracts to study the correlation between the two phytochemicals, that is, polyphenol and flavonoid, and their antioxidant ability in biological lipid substrates.

### 2.5. Preparation of Substrates

Three biological lipid substrates, LDL, brain and cholesterol, were chosen to analyze the potency of methanol extracts of the plant samples in inhibiting oxidation. Cholesterol was obtained from a commercial source and dissolved in ethanol to a known concentration and stored at 0°C until further use.

LDL was isolated by the modified method of Schlussel and Elstner [30]. 20 mL of blood was drawn from healthy human subjects by Laboratory Technician at University Health Centre, University of Mysore. Samples were added to Ethylenediaminetetraacetic acid (EDTA) vials and centrifuged at 2000 rpm for 20 minutes at 4°C. 3 mL of the plasma layer was separated and pipetted into 15 mL tubes. 1.5 g of KBr was added to the tubes and rocked until the KBr dissolved. 9 mL saline was added to the tubes and ultracentrifuged (Thermo Scientific, Pune) at 43,000 rpm for 3 h at 4°C. The bottom layer of the fractions was separated using a Pasteur pipette, dialyzed, and tested for LDL and protein content using standard kits. The content was diluted with phosphate buffered saline (PBS) to a known volume and stored in freezer until further use. Substrate equal to 1 mg protein concentration was taken for the experiment. Permission from Institutional Human Ethics Committee of the University of Mysore was obtained for the collection of blood samples from healthy subjects to isolate LDL (IHEC-UOM Number 36 Res/2013-14, date: 16/4/2013).

Brain was isolated from healthy male adult rats from Central Animal House of the University. It was washed in PBS (0.01 M, pH-7.4) and homogenized with six strokes of a smooth-walled, glass Remi homogenizer. The brain homogenate was stored in the freezer (4°C) until further use. The protein content of the homogenate was analyzed and substrate equal to 1 mg protein concentration was taken for the experiment. Permission from Institutional Animal Ethics Committee of the University of Mysore was obtained to isolate brain (UOM/IAEC/04/2013, date: 28/09/2013).

### 2.6. Estimation of TBARS

Lipid peroxide formation was measured by the modified method of Ohkawa et al. [31]. Substrates (cholesterol, LDL, and brain homogenate) were taken according to their protein content. Plant extracts were added to the substrates at different concentrations (300–500 microlitres of 1 mg/mL). Fenton's reagent was added to induce oxidation (500 *μ*L of 20 mM FeSO_4_ and 250 *μ*L of 20 mM H_2_O_2_) and the tubes were vortexed. The mixture was incubated for 2 hours at 50°C followed by addition of 1 mL of TBA (0.67%) and 1 mL of TCA (10%). The test tubes were incubated in boiling water bath for 30 minutes. After cooling, 3 mL of butanol was added to the tubes and vortexed. The butanol layer was separated and centrifuged at 3000 rpm for 10 min at 5°C and the absorbance was measured at 532 nm at room temperature:

(1) %  Inhibition  of  oxidation=O.D  of  control−O.D  of  sampleO.D  of  control×100.

## 3. Statistical Analysis

All the experiments were carried out in triplicates (*n* = 3). The correlation was determined by Pearson's product-moment correlation coefficient. Data was subjected to one-way ANOVA using SPSS software, 2011 version, (*p* ≤ 0.05).

## 4. Results and Discussion

Medicinal plants have undoubtedly been nature's fighters against oxidative stress related diseases. Apart from endogenous synthesis and production of antioxidants; supplementation of exogenous sources of antioxidants has become vital to curb the imbalance between antioxidant status and oxidative stress. Promoting the utilization of medicinal plants in treating degenerative diseases could be a novel and safer approach.

### 4.1. Antioxidant Activity of Medicinal Plants in Different Biological Substrates

**Figure 1:**
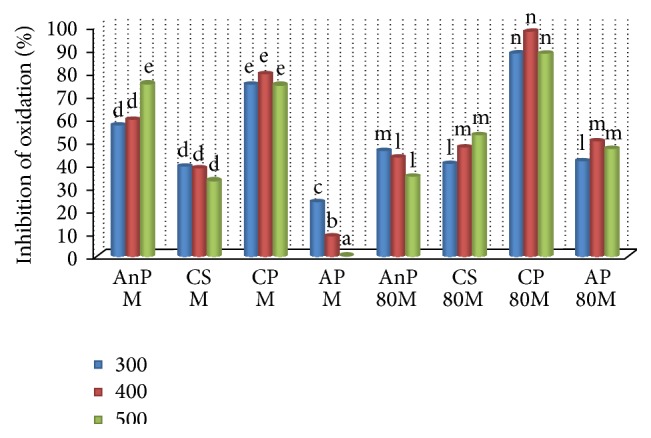
Inhibition of cholesterol oxidation by different plant extracts. AnP:* Andrographis paniculata*; CS:* Costus speciosus*; CP:* Canthium parviflorum*; AP:* Abrus precatorius*; M: methanol; 80M: 80% methanol; a, b, c,… represents significance of methanol extracts, l, m, n,… represents significance of 80% methanol extracts (*p* ≤ 0.05). Values are the mean of triplicates (*n* = 3).

The antioxidant activity of the samples in cholesterol is given in Figure 1. It is evident that the extracts of the plant samples have shown varied inhibitory activity; that is, the activity did not increase with increasing concentrations. It was observed that both M and 80M of CP showed significantly higher activity compared to other plant samples (*p* ≤ 0.05). Also, maximum activity was exhibited by both the extracts of CP at lower concentration at 400 *μ*g than at 500 *μ*g (80M: 97.55% and M: 79.2% at 400 *μ*g and 80M: 88.07% and M: 74.31% at 500 *μ*g, resp.). The oxidation inhibitory potential of CS M, AP M, and AnP 80M decreased with increasing concentration and AP showed significantly the least activity among the sample extracts (*p* ≤ 0.05). The antioxidant activity of CP could be due to the presence of lipid soluble phytochemicals such as *α*-tocopherol (164.6 ± 22.10 mg/100 g dry basis) and *β*-carotene (1060 ± 0.6 *μ*g/100 g dry basis) in higher amounts [27]. The protective effect of tocopherols against cholesterol oxidation is well known and reported in several studies [16, 32]. CP has also exhibited good antioxidant activity against oxidation of biological substrates such as microsomes and RBC [24, 27]. Decrease in antioxidant activity with increasing concentrations could be due to the prooxidant activity of some phytochemicals at higher concentrations [33].

**Figure 2:**
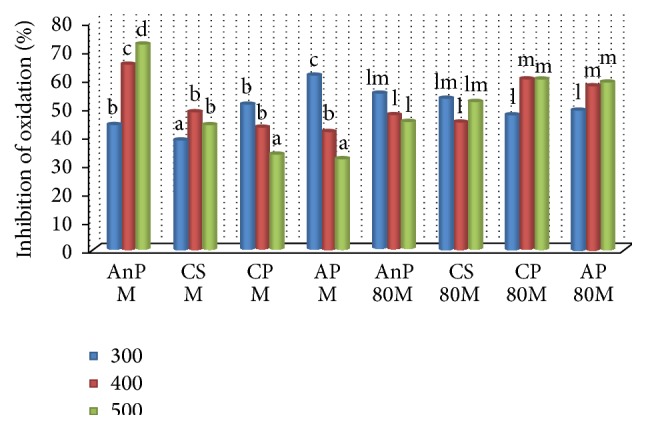
Inhibition of LDL oxidation by different plant extracts. AnP:* Andrographis paniculata*, CS:* Costus speciosus*, CP:* Canthium parviflorum*, AP:* Abrus precatorius*; M: Methanol, 80M: 80% Methanol; a, b, c,… represent significance of methanol extracts; l, m, n,… represent significance of 80% methanol extracts (*p* ≤ 0.05). Values are the mean of triplicates (*n* = 3).

The antioxidant activity of the samples in LDL is given in Figure 2. Most of the plant extracts exhibited a varied trend in LDL as observed in the figure, that is, the inhibitory activity did not follow an increasing trend. However, AnP M (72.15%) showed significantly higher antioxidant potential than other extracts (*p* ≤ 0.05). A decreasing trend was observed in CP M and AP M with the lowest antioxidant activity. All four 80M extracts showed close values of antioxidant potential ranging between 44 and 59% and there was no significant difference observed between the extracts (*p* ≥ 0.05). AnP is a rich source of polyphenol (3.21 ± 0.10 g/100 g), flavonoid (1.50 ± 0.16 mg/100 g), and glutathione (824 ± 28.84 mmol/100 g) [27]. Also, the polyphenol and flavonoid content of AnP M showed good correlation with the antioxidant activity compared to other extracts (*r* = 0.948, *p* ≤ 0.01). The protective effect of polyphenols, flavonoids, and tocopherol against oxidation of LDL has been reported [34–36].

**Figure 3:**
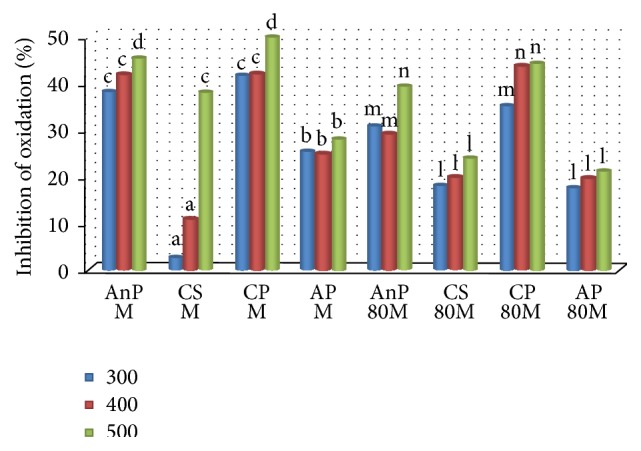
Inhibition of lipid oxidation in brain homogenate by different plant extracts. AnP:* Andrographis paniculata*; CS:* Costus speciosus*; CP:* Canthium parviflorum*; AP:* Abrus precatorius*; M: methanol; 80M: 80% methanol; a, b, c,… represent significance of methanol extracts; l, m, n,… represent significance of 80% methanol extracts (*p* ≤ 0.05). Values are the mean of triplicates (*n* = 3).

The antioxidant activity of the samples in brain homogenate is given in Figure 3. Methanol extracts of CP (49.55%) and AnP (45.17%) showed significantly high inhibition of oxidation in brain homogenate (*p* ≤ 0.05). The trend of inhibition of oxidation in brain homogenate was highly comparable to that of cholesterol wherein both the extracts of CP showed higher inhibition of oxidation than other extracts. CS 80M and AP 80M showed significantly the least activity in brain (*p* ≤ 0.05). All extracts showed an increase in antioxidant activity with increasing concentrations except AnP 80M and AP M. There are no reported studies on the protective effect of CP on brain homogenate; however, CP has been reported to inhibit oxidation of RBC and microsomes [27]. In a study, the protective effect of methanol extract of AnP leaves was evident by decreased tissue malondialdehyde (MDA) levels and increased SOD levels owing to its antioxidant and cerebroprotective activity against cerebral infarction in Type II diabetic animal model [37].

### 4.2. Correlation between the Polyphenol, Flavonoid Content, and Antioxidant Activity

The polyphenol and flavonoid content of all the extracts were analyzed. It was observed that AnP and AP had a higher polyphenol and flavonoid content in the methanol extract than CS and CP, whereas AP and CS had higher polyphenol content; CP and AP had higher flavonoid content in 80% methanol extract.

Antioxidant activity of an extract can be correlated to its phytochemical constituents by Pearson's product-moment correlation coefficient. Accordingly, the polyphenol and flavonoid content were correlated with antioxidant activity in all three substrates (Tables 1 [Table tab2]–3).

In cholesterol, AnP M had the highest polyphenol content and exhibited good correlation (0.816, *p* ≤ 0.01) compared to other extracts. This effect could be due to the presence of polyphenols (3.21 ± 0.10 g/100 g dry basis) and flavonoids (1.50 ± 0.16 mg/100 g dry basis) in higher concentration in AnP than other medicinal plants. The presence of higher amounts of Glutathione (824 ± 28.84 mmol/100 g dry basis) and saponins in AnP M might have contributed to the total antioxidant activity [27]. Extracts of AnP M, CS 80M, and AP 80M showed a positive correlation. Although CP M showed higher inhibition of oxidation, correlation between the flavonoid and polyphenol content did not reach statistical significance (*p* ≥ 0.01), suggesting the role of other phytochemicals. The presence of flavonoids, polyphenols, and andrographolide diterpenoids in AnP has been reported [38].

In LDL, as a substrate, extracts of AnP M (*r* = 0.948, *p* ≤ 0.01), AP 80M (*r* = 0.840, *p* ≤ 0.01), and CP 80M (*r* = 0.776, polyphenols, and *r* = 0.800, flavonoids, *p* ≤ 0.01) showed a higher correlation than other extracts. AnP M showed better correlation in LDL than other substrates. This could be due to the compositional differences between the substrates. AP 80M had the highest amount of polyphenol and flavonoid content (120 ± 0 mg/g and 1.69 ± 0.02 mg/g, resp.) among all extracts and the antioxidant activity (48.65–58.67%) correlated well with the presence of these phytochemicals in greater amount. Methanol extracts of AnP and CS and 80% methanol extracts of CP and AP showed positive correlation in LDL.

Highest correlation was observed between flavonoid, polyphenol content, and the antioxidant activity by CS M (*r* = 0.977, polyphenols and *r* = 0.949, flavonoids, *p* ≤ 0.01) in brain homogenate compared to other substrates and extracts. CS M showed a significant increasing trend in antioxidant activity and good correlation at very low concentration of polyphenols (51.66 ± 2.35 mg/g) and flavonoids (0.04 ± 0 mg/g) compared to other extracts. As observed in cholesterol, though CP M showed the highest antioxidant activity, there was no correlation observed between the antioxidant activity and its flavonoid and polyphenol content signifying contribution towards the antioxidant activity by other phytochemicals. The presence of phenolic compounds in CS has been reported [39]. Though AnP and AP had a higher polyphenol and flavonoid content than CS, the correlation did not reach statistical significance (*p* ≥ 0.01).

## 5. Conclusion

This research paper is the first comparative* ex vivo* study reporting the antioxidant potency of* Andrographis paniculata*,* Canthium parviflorum*,* Costus speciosus*, and* Abrus precatorius *in biological lipid substrates. Among the four samples studied,* Canthium parviflorum* and* Andrographis paniculata* exhibited high antioxidant potential against the three biological lipid substrates owing to the presence of phytochemicals such as polyphenols, flavonoids, glutathione, *α*-tocopherol, and *β*-carotene. Further studies are needed to confirm these observations using* in vivo* models simulating pathophysiology of neurodegenerative and atherogenic diseases.

## Figures and Tables

**Table 1:** Correlation between the antioxidant activity of plant extracts against cholesterol oxidation and the content of polyphenol and flavonoid in the extracts.

	M	*r*-value	80M	*r*-value
Polyphenol (mg/g)				
AnP	88.33 ± 2.35	0.816	41.66 ± 2.35	−0.617
CS	51.66 ± 2.35	−0.459	63.33 ± 2.35	0.652
CP	46.66 ± 4.71	−0.040	55.00 ± 0	−0.040
AP	83.33 ± 9.42	−0.968	120.00 ± 0	0.508
Flavonoid (mg/g)				
AnP	1.87 ± 0.07	0.816	0.96 ± 0.11	−0.617
CS	0.04 ± 0	−0.439	0.40 ± 0.06	0.655
CP	0.59 ± 0.01	−0.02	1.09 ± 0.01	−0.000
AP	2.58 ± 0.10	−0.973	1.69 ± 0.02	0.508

Values are mean of triplicates (*n* = 3); M: methanol extract; 80M: 80% methanol extract; *r*-value: *p* ≤ 0.01; AnP: *Andrographis paniculata*; CS: *Costus speciosus*; CP: *Canthium parviflorum*; AP: *Abrus precatorius*.

**Table 2 tab2:** Correlation between the antioxidant activity of plant extracts against oxidation of LDL and the content of polyphenol and flavonoid in the extracts.

	M	*r*-value	80M	*r*-value
Polyphenol (mg/g)				
AnP	88.33 ± 2.35	0.948	41.66 ± 2.35	−0.761
CS	51.66 ± 2.35	0.414	63.33 ± 2.35	−0.034
CP	46.66 ± 4.71	−0.958	55.00 ± 0	0.776
AP	83.33 ± 9.42	−0.934	120.00 ± 0	0.840
Flavonoid (mg/g)				
AnP	1.87 ± 0.07	0.948	0.96 ± 0.11	−0.761
CS	0.04 ± 0	0.501	0.40 ± 0.06	−0.076
CP	0.59 ± 0.01	−0.957	1.09 ± 0.01	0.800
AP	2.58 ± 0.10	−0.940	1.69 ± 0.02	0.840

Values are mean of triplicates (*n* = 3); M: methanol extract; 80M: 80% methanol extract; *r*-value: *p* ≤ 0.01; AnP: *Andrographis paniculata*; CS: *Costus speciosus*; CP: *Canthium parviflorum*; AP: *Abrus precatorius*.

**Table 3:** Correlation between the antioxidant activity of plant extracts against lipid oxidation in brain homogenate and the content of polyphenol and flavonoid in the extracts.

	M	*r*-value	80M	*r*-value
Polyphenol (mg/g)				
AnP	88.33 ± 2.35	0.041	41.66 ± 2.35	0.718
CS	51.66 ± 2.35	0.977	63.33 ± 2.35	0.706
CP	46.66 ± 4.71	0.584	55.00 ± 0	0.591
AP	83.33 ± 9.42	0.700	120.00 ± 0	0.720
Flavonoid (mg/g)				
AnP	1.87 ± 0.07	0.041	0.96 ± 0.11	0.718
CS	0.04 ± 0	0.949	0.40 ± 0.06	0.701
CP	0.59 ± 0.01	0.566	1.09 ± 0.01	0.608
AP	2.58 ± 0.10	0.683	1.69 ± 0.02	0.720

Values are mean of triplicates (*n* = 3); M: methanol extract; 80M: 80% methanol extract; *r*-value: *p* ≤ 0.01; AnP: *Andrographis paniculata*; CS: *Costus speciosus*; CP: *Canthium parviflorum*; AP: *Abrus precatorius*.

## References

[1] Birben E., Sahiner U. M., Sackesen C., Erzurum S., Kalayci O. (2012). Oxidative stress and antioxidant defense. *World Allergy Organization Journal*.

[2] Butterfield D. A., Lange M. L. B., Sultana R. (2010). Involvements of the lipid peroxidation product, HNE, in the pathogenesis and progression of Alzheimer's disease. *Biochimica et Biophysica Acta (BBA)—Molecular and Cell Biology of Lipids*.

[3] Reiter R. J. (1995). Oxidative processes and antioxidative defense mechanisms in the aging brain. *The FASEB Journal*.

[4] Gutteridge J. M. C. (1995). Lipid peroxidation and antioxidants as biomarkers of tissue damage. *Clinical Chemistry*.

[5] Yoshida Y., Umeno A., Shichiri M. (2013). Lipid peroxidation biomarkers for evaluating oxidative stress and assessing antioxidant capacity *in vivo*. *Journal of Clinical Biochemistry and Nutrition*.

[6] Niki E., Yoshida Y., Saito Y., Noguchi N. (2005). Lipid peroxidation: mechanisms, inhibition, and biological effects. *Biochemical & Biophysical Research Communications*.

[7] Stocker R., Bowry V. W., Frei B. (1991). Ubiquinol-10 protects human low density lipoprotein more efficiently against lipid peroxidation than does alpha-tocopherol. *Proceedings of the National Academy of Sciences of the United States of America*.

[8] Esterbauer H., Rotheneder M., Striegl G. (1989). Vitamin E and other lipophilic antioxidants protect LDL against oxidation. *Lipid/Fett*.

[9] Fuhrman B., Aviram M. (2001). Flavonoids protect LDL from oxidation and attenuate atherosclerosis. *Current Opinion in Lipidology*.

[10] Aviram M., Fuhrman B. (2002). Wine flavonoids protect against LDL oxidation and atherosclerosis. *Annals of the New York Academy of Sciences*.

[11] Milde J., Elstner E. F., Grabmann J. (2007). Synergistic effects of phenolics and carotenoids on human low-density lipoprotein oxidation. *Molecular Nutrition & Food Research*.

[12] Zaidi S. M. K. R., Banu N. (2004). Antioxidant potential of vitamins A, E and C in modulating oxidative stress in rat brain. *Clinica Chimica Acta*.

[13] Kamat J. P., Devasagayam T. P. A. (1999). Nicotinamide (vitamin B3) as an effective antioxidant against oxidative damage in rat brain mitochondria. *Redox Report*.

[14] Galli R. L., Shukitt-Hale B., Youdim K. A., Joseph J. A. (2002). Fruit polyphenolics and brain aging. *Annals of the New York Academy of Sciences*.

[15] Ho J. H., Chang Y. L. (2004). Protective effects of quercetin and vitamin C against oxidative stress-induced neurodegeneration. *Journal of Agricultural and Food Chemistry*.

[16] Xu Z., Hua N., Godber J. S. (2001). Antioxidant activity of tocopherols, tocotrienols, and *γ*-oryzanol components from rice bran against cholesterol oxidation accelerated by 2,2′-azobis(2-methylpropionamidine) dihydrochloride. *Journal of Agricultural and Food Chemistry*.

[17] Kumar P. S., Sucheta S., Deepa V. S., Selvamani P., Latha S. (2008). Antioxidant activity in some selected Indian medicinal plants. *African Journal of Biotechnology*.

[18] Kumari A., Kakkar P. (2008). Screening of antioxidant potential of selected barks of indian medicinal plants by multiple *in vitro* assays. *Biomedical and Environmental Sciences*.

[19] Pulla Reddy A. C., Lokesh B. R. (1994). Effect of dietary turmeric (*Curcuma longa*) on iron-induced lipid peroxidation in the rat liver. *Food and Chemical Toxicology*.

[20] Reddy P. V., Urooj A., Kumar A. (2005). Evaluation of antioxidant activity of some plant extracts and their application in biscuits. *Food Chemistry*.

[21] Roy L. G., Arabshahi-Delouee S., Urooj A. (2010). Antioxidant efficacy of mulberry (*Morus indica* L.) leaves extract and powder in edible oil. *International Journal of Food Properties*.

[22] Reddy P. V., Sahana N., Urooj A. (2012). Antioxidant activity of *Aegle marmelos* and *Psidium guajava* leaves. *International Journal of Medicinal and Aromatic Plants*.

[23] Poodineh M. (2013). *Effect of fruit peel, spice and herb extracts on the oxidative stability of Sunflower oil [Ph.D. dissertation]*.

[24] Reddy P. V., Mahalingu S., Urooj A. (2014). *Canthium parviflorum* leaves: antioxidant activity in food and biological systems, pH, and temperature stability. *Chinese Journal of Biology*.

[25] Palvai V. R., Mahalingu S., Urooj A. (2014). *Abrus precatorius* leaves: antioxidant activity in food and biological systems, pH, and temperature stability. *International Journal of Medicinal Chemistry*.

[26] Palvai V. R., Urooj A. (2014). Inhibition of 3-hydroxy-3-methylglutaryl coenzyme A reductase (*ex vivo*) by *Morus indica* (Mulberry). *Chinese Journal of Biology*.

[27] Pai Kotebagilu N., Reddy Palvai V., Urooj A. (2014). Protective effect of selected medicinal plants against hydrogen peroxide induced oxidative damage on biological substrate. *International Journal of Medicinal Chemistry*.

[28] Slinkard K., Singleton V. L. (1967). Total phenol analysis, automation and comparison with manual methods. *American Journal of Enology and Viticare*.

[29] Miliauskas G., Venskutonis P. R., Van Beek T. A. (2004). Screening of radical scavenging activity of some medicinal and aromatic plant extracts. *Food Chemistry*.

[30] Schlussel E. H., Elstner E. F. (1996). Desialylation of low density lipoprotein—metabolic function versus oxidative damage?. *Zeitschrift fur Naturforschung C*.

[31] Ohkawa H., Ohishi N., Yagi K. (1979). Assay for lipid peroxides in animal tissues by thiobarbituric acid reaction. *Analytical Biochemistry*.

[32] Grau A., Codony R., Grimpa S., Baucells M. D., Guardiola F. (2001). Cholesterol oxidation in frozen dark chicken meat: influence of dietary fat source, and *α*-tocopherol and ascorbic acid supplementation. *Meat Science*.

[33] Bouayed J., Bohn T. (2010). Exogenous antioxidants—double-edged swords in cellular redox state: health beneficial effects at physiologic doses versus deleterious effects at high doses. *Oxidative Medicine and Cellular Longevity*.

[34] Osada K., Takahashi M., Hoshina S., Nakamura M., Nakamura S., Sugano M. (2001). Tea catechins inhibit cholesterol oxidation accompanying oxidation of low density lipoprotein *in vitro*. *Comparative Biochemistry and Physiology Part C: Toxicology & Pharmacology*.

[35] Vaya J., Mahmood S., Goldblum A. (2003). Inhibition of LDL oxidation by flavonoids in relation to their structure and calculated enthalpy. *Phytochemistry*.

[36] Mutalib M. S. A., Khaza'ai H., Wahle K. W. J. (2003). Palm-tocotrienol rich fraction (TRF) is a more effective inhibitor of LDL oxidation and endothelial cell lipid peroxidation than *α*-tocopherol *in vitro*. *Food Research International*.

[37] Radhika P., Annapurna A., Nageswara Rao S. (2012). Immunostimulant, cerebroprotective & nootropic activities of *Andrographis paniculata* leaves extract in normal & type 2 diabetic rats. *The Indian Journal of Medical Research*.

[38] Rao Y. K., Vimalamma G., Rao C. V., Tzeng Y.-M. (2004). Flavonoids and andrographolides from *Andrographis paniculata*. *Phytochemistry*.

[39] Nehete J., Bhatia M., Narkhede M. (2010). *In vitro* evaluation of antioxidant activity and phenolic content of *Costus speciosus* (Koen ex.Retz.) Sm. *Iranian Journal of Pharmaceutical Research*.

